# Validation of the Neonatal Satisfaction Survey (NSS-8) in six Norwegian neonatal intensive care units: a quantitative cross-sectional study

**DOI:** 10.1186/s12913-018-3031-z

**Published:** 2018-03-27

**Authors:** Inger Hilde Hagen, Marit Følsvik Svindseth, Erik Nesset, Roderick Orner, Valentina Cabral Iversen

**Affiliations:** 1 NTNU Norwegian University of Science and Technology, Aalesund, Postbox 1517, 6025 Aalesund, Norway; 2 College of Social Science, University of Lincoln, Brayford Pool, Lincoln, Lincolnshire LN6 7TS UK; 3 St Olav’s University Hospital HF, Tiller District Psychiatric Center, Trondheim, Norway; 4 NTNU Norwegian University of Science and Technology, Faculty of Medicine and Health Science, 7491 Trondheim, Norway

**Keywords:** Parents’ satisfaction, NICU, Health care, Neonatal, Survey, Validity, Reliability

## Abstract

**Background:**

The experience of having their new-borns admitted to an intensive care unit (NICU) can be extremely distressing. Subsequent risk of post-incident-adjustment difficulties are increased for parents, siblings, and affected families. Patient and next of kin satisfaction surveys provide key indicators of quality in health care. Methodically constructed and validated survey tools are in short supply and parents’ experiences of care in Neonatal Intensive Care Units is under-researched. This paper reports a validation of the Neonatal Satisfaction Survey (NSS-8) in six Norwegian NICUs.

**Methods:**

Parents’ survey returns were collected using the Neonatal Satisfaction Survey (NSS-13). Data quality and psychometric properties were systematically assessed using exploratory factor analysis, tests of internal consistency, reliability, construct, convergent and discriminant validity. Each set of hospital returns were subjected to an apostasy analysis before an overall satisfaction rate was calculated.

**Results:**

The survey sample of 568 parents represents 45% of total eligible population for the period of the study. Missing data accounted for 1,1% of all returns. Attrition analysis shows congruence between sample and total population. Exploratory factor analysis identified eight factors of concern to parents,“Care and Treatment”, “Doctors”, “Visits”, “Information”, “Facilities”, “Parents’ Anxiety”, “Discharge” and “Sibling Visits”. All factors showed satisfactory internal consistency, good reliability (Cronbach’s alpha ranged from 0.70–0.94). For the whole scale of 51 items α 0.95. Convergent validity using Spearman’s rank between the eight factors and question measuring overall satisfaction was significant on all factors. Discriminant validity was established for all factors. Overall satisfaction rates ranged from 86 to 90% while for each of the eight factors measures of satisfaction varied between 64 and 86%.

**Conclusion:**

The NSS-8 questionnaire is a valid and reliable scale for measuring parents’ assessment of quality of care in NICU. Statistical analysis confirms the instrument’s capacity to gauge parents’ experiences of NICU. Further research is indicated to validate the survey questionnaire in other Nordic countries and beyond.

## Background

Approximately 10% of all new-borns in Norway require advanced health care soon after birth [1]. For parents, the experience of having their new-born infant admitted to an intensive care unit (NICU) can be extremely distressing. Subsequent risk of post-incident-adjustment difficulties are increased for parents, siblings, and affected families. A protective factor is a positive association between parent satisfaction with neonatal health care and parents ability to provide need-based care for their child(ren) [2–4]. When this includes delivering treatment to infants, better compliance is achieved and sustained by parents who report a higher level of satisfaction with neonatal health care [5–7].

Systematic screening of patient satisfaction should therefore be an essential part of quality-monitoring and quality improvement initiatives [8, 9]. By monitoring the patients’ experiences, hospital units gather data which can be used to guide changes towards improved service provision, not only during the inpatient phase but also, by implication, after discharge [8].

Several instruments have been developed to gauge parent satisfaction with care in ICUs [5, 10–12], including assessment of mothers satisfaction with intrapartum care and childbirth [6]. Instruments developed to measure parent satisfaction with care in neonatal ICU (NICU) are few in number [13, 14], and none are developed for use in Scandinavia. Latour (2012) recently developed such a questionnaire in the Netherlands, and Sawyer (2014) has done the same with a focus on care provided for very preterm infants in the U.K. With these considerations in mind, there is a pressing need to develop an instrument which measures parent satisfaction with care in NICU. Our first endeavour is to validate such an instrument for use in Scandinavia.

NICUs in Norway treat approximately 6000 children each year. Of these, about 60% are born at gestation (age ≥ 37 weeks), 22% are born between 34 and 37 weeks, and about 18% are born at 34 weeks or less [15].

Norwegian national guidelines for health personnel aim to help parents give optimal care for their children, especially under circumstances considered to be personally challenging or difficult for families. Parents have established legal rights to be with their children when they so wish, and to this end, they receive mandatory information pertinent to their child’s health status. There is also explicit recognition that parents have a right to participate actively in decision-making processes about their child’s health care [16, 17].

The aim of this study is to validate the Neonatal Satisfaction Survey (NSS-13) [18] in six Norwegian NICUs. The survey measures parents’ level of satisfaction with the care provided for their premature or sick infant. The selected NICUs are in different regional hospitals spanning a wide geographical area with a diversity of urban and rural catchment areas.

## Methods

### Questionnaire development

In a former study [18], the Neonatal Satisfaction Survey (NSS-13) questionnaire was developed by a phased structured process intended to deliver a valid and discriminating survey tool. To establish convergent/concurrent and discriminant validity, the process started with a literature review which sought to extract “gold standard” for quality of care in NICUs.

In preparation for the pilot study [18], we included questions from Garrat et al.’s earlier informal survey [5] which included criteria for quality of hospital health care established by government decree in Norway [19].

Content validity (or face validity) refers to expert opinions on whether the scale items represent the proposed domains of concepts that the questionnaire is intended to measure. In order to establish what these may be, we convened a focus-group with participants selected from health personnel with relevant experience and expertise (*n* = 18). Also included were parents who had experiences from NICU (*n* = 10). They were asked to review a list of questions for relevance, clarity, and readability as well as to propose additional questions.

Two control questions, with an identical rating scale, measuring overall satisfaction were added to advance the process of developing the NSS-13 [20–22]. Content validity was tested on a small sample of 105 respondents by using the NSS-13 (pilot study) [18].

Having collated this baseline information, the next step in this project was to collect data required for a formal evaluation of the NSS-13 using factor analytic techniques. The power calculation was based on the previous studies. Hair et al.’s. [23] recommend a ratio of five observations per variable.

### Measurement

The NSS-13 questionnaire contains 69 items derived from 13 categories or themes relevant to parents’ satisfaction with care provided in NICUs. Themes are Staff, Admission, Nursing Personnel, Anxiety, Siblings and Other Next of Kin, Information, Time Out, Doctors, Facilities, Nutrition, Preparation for Discharge, Trust, and Visitors. An English language version of the NSS-13 was developed by translating to English and then back to Norwegian by professional translators.

### Participant procedure

Participants of this study were Norwegian or English-speaking parents admitted to one of the six NICUs whose stays had lasted for more than two days. The infants’ gestation age ranged from 24 to 42 weeks. The NICUs admitted also infants up to 3-months after birth. The self-administrated questionnaire was given to the parents a few days before they were due to leave hospital, in order to facilitate a calm atmosphere. Parents with multiple births received only one questionnaire and those whose children passed away while in the unit were excluded from this study.

### Data collection

The first author contacted the heads of 12 NICUs in Norway to invite their participation in the study. Six of the units could not participate because of an overload of studies at the time. Agreement was obtained from six units, each of which would collect at least 100 completed questionnaires. These six units are in a geographical spread area, with a variety of universities and local hospitals (level 2–3), and can therefore represent a valid selection of the NICU’s in Norway. Once this had been agreed, the multicentre prospective cohort study could proceed and was conducted between September 2015 and October 2016.

Participating NICUs varied in size from 6 to 21 beds (mean 12.5) and treated from 253 to 500 patients each year. Two NICUs are university hospitals, and the rest have regional or local catchment areas. Three units treated infants with ≥GA 23, and the rest cared for newborn infant with GA 26–30.

The first author introduced the study for the nurses in the units, and three research assistants were responsible for questionnaire distribution and collection. The research protocol to be followed was distributed to all unit nurses and were placed in a prominent position within each unit’s nurses’ station. During the collection of data period, the first author had regular contact with research assistants by telephone and email. Some units were also visited during the date collection phase.

As discharge approached, the research assistant contacted the infant’s next of kin to secure their informed consent to take part in this study. The research assistant left a copy of the self-report questionnaire with parents who had agreed to take part. Confidentiality arrangements were explained, as was the protocol, that no completed questionnaires would be read by anyone working at the unit. The participants used about 30 min to complete the answering of the survey.

Respondents provided demographic information about themselves and their infant (Tables 1 and 2). To avoid sampling errors, we carried out an attrition analysis for each hospital because of the inclusion criteria (Table 3).

**Table 1:** Demographics of parents participating in the Neonatal Satisfaction Study

Variables	Mother (*N* = 312)	Father (*N* = 256)	X^2^-test *p*-value	Total (*N* = 568)
Age at admission, Mean (SD)	30.09 (5.50)	33.10 (6.94)		
	N(%)	N(%)		N
Marital status			0.599	
Married/In relationship	300 (96.5)	247 (97.2)		547
Divorced/Single parent	11 (3.5)	7 (2.8)		18
Total	311 (100)	254 (100)		654
Level of education			0.013	
Higher education > 4 years	76 (24.4)	50 (19.7)		126
Higher education < 4 years	108 (34.6)	66 (26.0)		174
College	113 (36.2)	126 (49.6)		239
Grammar school	15 (4.8)	12 (4.7)		27
Total	312 (100)	254 (100)		566
Work status			0.001	
In paid work	217 (69.6)	232 (90.6)		449
Not paid work/education	95 (30.4)	24 (9.4)		119
Total	312 (100)	256 (100)		568
Travel time to hospital			0.404	
Less than 1 h	156 (50.0)	137 (53.5)		294
More than 1 h	156 (50.0)	119 (46.5)		275
	312 (100)	256 (100)		

Cross-tabulation and Chi-square tests

**Table 2:** Demographics of the parents’ infant (*N* = 352) participating in the Neonatal Satisfaction Study

Variables	Total (%)
Was your child premature or born at term?	
Premature (< 37 weeks)	245 (70)
Born at term (≥ 37)	107 (29)
Missing	2 (1)
Total	352
Multiple birth	29 (0.82)
Length of stay	
< 1 week	93 (29)
1–2 weeks	104 (32)
2–4 weeks	58 (18)
> 4 weeks	62 (19)
Missing	8 (2)
Total	325
Parents’ evaluation of the child’s health (*N* = 568)	
Good	532 (94)
Bad	22 (4)
Missing	9 (2)
Total	568

**Table 3:** Dropout calculation and average of the selection and population from NICUs in 6 hospitals

	Dropout calculation and average of the selection and population from 6 hospitals							
	Unit 1 (level 2)	Unit 2 (level 3)	Unit 3 (level 2)	Unit 4 (level 3)	Unit 5 (level2)	Unit 6 (level 2)	Sum population	Sum selection^a^
	N(%)	N(%)	N(%)	N(%)	N(%)	N(%)	N(%)	
Number of patients admitted per year (2015) Population	330	439	253	255	196	232	1705	
Number of infants admitted during the collection period Population	150	322	141	226	169	167	1175	
Quantity after inclusion criteria Population	95 infants thereof 5 TV = 89 families	203 infants thereof 11 TV = 192 families	85 infants thereof 6 TV = 79 families	139 infants thereof 11 TV = 128 families	132 infants thereof 4 TV = 128 families	109 infants thereof 4 TV = 105 families	763 infants thereof 41 TV = 722 families	
Number of families who have answered	59	64	40	53	56	53		325
Answers %	66%	33%	51%	41%	44%	50%		45%
Gestational age Population								
24–28 weeks(%)	5(5.2) (1TV-par)	15(7.3) (2TV)	6(7.0) (2TV)	9(6.4) (1TV)	6(4.5)	2(1.8)	43(5.6)	
29–33 weeks(%)	14(14.7) (2 TV)	66(32.5) (7TV)	18(21.1) (2TV)	30(21.5) (3TV)	19(14.3) (2TV)	20(18.3) (2TV)	167(21.8)	
34–37 weeks(%)	28(29.4) (2 TV)	60(29.5) (2TV)	33(38.8) (2TV)	44(31.6) (7TV)	33(25) (3TV)	35(32.1) (2TV)	233(30.5)	
38–42 weeks(%)	47(49.4)	62(30.5)	27(31.7)	56(40.2)	70(53)	50(45.8)	312(40.8)	
> 42 weeks(%)	1(1.0)	0	1(1.1)	0	4(3,0)	2(1.8)	8(1)	
Total number of infant Population	95(100)	203(100)	85(100)	139(100)	132(100)	109(100)	763(100)	
Total number of infant Selection	63(66%)	69(33%)	44(51%)	59(42%)	58(44%)	54(49%)		347(45%)
Gestational age Selection								
24–28 weeks(%)	4 (6,8)	8(12.5)	1(2.5)	5(9.4)	5(8.9)	1(1.8)		24(7.4)
29–33 weeks(%)	11(18,6)	19(29.6)	16(40)	11(20.7)	7(12.5)	19(35.8)		83(25.6)
34–37 weeks(%)	19(32.2)	23(35.3)	12(30)	13(24.5)	12(21.4)	19(35.8)		98(30,3)
38–42 weeks(%)	22(37.3)	12(18.7)	10(25)	20(37.7)	30(53.5)	11(20.7)		105(32.5)
> 42 weeks(%)	3(5.1)	2(3.1)	1(2.5)	2(3.7)	2(3.5)	3(5.6)		13(4)
Total families	59(100)	64(100)	40(100)	51 (96.2) 2 missing	56 (100)	53 (100)		323(99.3)
Length of stay Population								
< 1 week(%)	32(35.9)	59(30.7)	24(30.3)	53(41.4)	60(46.8)	62(59)	290(40.2)	
1–2 weeks(%)	39(43.8)	72(37.5)	28(35.4)	29(22.6)	38(29.6)	22(20,9)	228(31.6)	
2–4 weeks(%)	13(14.6)	36(18.7)	18(22.7)	25(19.5)	16(12.5)	18(17.1)	126(17.4)	
> 4 weeks(%)	5(5.6)	25(13)	9(11.3)	21(16.4)	14(10.9)	3(2.8)	77(10.6)	
Totalt families	89 (100)	192(100)	79(100)	128(100)	128(100)	105 (100)	721	
Length of stay Selection								
< 1 week(%)	21(35.6)	19(29.6)	6(15)	20(37.7)	20(35.7)	10(18.8)		96(30.2)
1–2 weeks(%)	20(33.9)	17(26.5)	16(40)	12(22.6)	19(33.9)	18(33.9)		102(32.1)
2–4 weeks(%)	9(15.2)	14(21.8)	6(15)	10(18.8)	4(7.1)	15(28.3)		58(18.2)
> 4 weeks(%)	9(15.2)	11(17.1)	11(27.5)	9(16.9)	12(21.4)	9(16.9)		61(19.2)
Total	59(100)	61(95.3)	39(97.5)	51(96.2)	55(98.2)	52(98.1)		317(97.5)
		3 missing	1 missing	2 missing	1 missing	1 missing		

Dichotomization of the parents’ perception of their child’s health is set to good (excellent, very good, and good) and to bad (from fairly good to bad)

^a^The different total Ns are explained by different populations as shown in the first column in the table

### Data analysis

Descriptive statistics were first conducted. All items used in the survey were first analysed by the descriptive information given. Mother and father demographic differences were analysed with the Pearson chi-square test, (*p* value set to ≤0.05). Correlations were tested by using Spearman rho. All significant tests are two-tailed.

Factor analysis was then used for data reduction in order to assess the underlying dimensions - or factors - of the questionnaire. The factor extraction was based on the principal component method, using the total variance of all variables (23). To assess the appropriateness of the factor analysis regarding sampling adequacy (high level of multicollinearity), the Kaiser-Meyer-Olkin (KMO) statistic and the Bartlett’s test of Sphericty were used. The KMO varies from 0 to 1, and predicts the likelihood of the data to factor well based on correlations and partial correlations. KMO should be larger than 0.5 (23). The Bartlett’s test of Sphericity tests the null hypothesis that the intercorrelations matrix stems from a population in which the variables have no correlations (23).

The factor loading of variables on a particular factor indicates the correlation between the variable and the factor, and should be higher than 0.3 in order to contribute to the overall KMO. Variables with factor loadings below 0.30 were eliminated.

Initially, we decided to remove 12 of the 15 items that did not significantly correlate with other questions. All six questions about nutrition were omitted from the factor analysis along with two questions containing staff caring about the infants’ stress and pain, unexpected waits (latency) in the unit, personnel communicating hope, children given the wrong treatment, and the parents’ need for follow-up regarding their own reactions. The three other questions regarding two items related to whether the parents were offered or needed a break from the NICU and information about the result of tests seem to be too clinically important to be removed at this stage.

Through a process of different exploratory factor analyses, we ended up with a final solution with eight factors. The basis for finding this number of factors was the Latent Root Criterion (eigenvalues larger than 1), which is a measure of the variance explained of each factor compared to the total variance. The factor solution used the orthogonal rotation method Varimax (23).

To establish questionnaire reliability (repeatability, stability and internal consistency), Cronbach’s alpha, average variance extracted, and inter-subscale correlations were calculated. The total score might be biased, especially for small sample sizes, because the item itself is included in the total score [24]. To reduce bias, a corrected item-total correlation was also calculated. This is a correlation of individual questions with the scale total omitted; a coefficient of around 0.3 is considered acceptable [14]. To assess discriminant validity we applied the Fornell-Larcker criterion [25], where the average variance-extracted values (AVE) for any two latent constructs are compared with the square of the correlation estimate between these two constructs. Discriminant validity is present when AVE is larger than the squared correlations [25]. The items used for the factor modelling are originally measured on a 1–5 scale. In order to present results of the different factors in percentage rates, they have been transformed to a 0–100 scale.

### Ethics

The study is conducted according to the Helsinki declaration. This project was first presented to the Regional Committees for Medical and Health Research Ethics which reported that the project was outside its mandate (2015/386). The project is approved from the Norwegian Data Protection Officials. All the respondents were asked to give oral and written consent to participate after having read an information sheet about the study which emphasized that participation was voluntary and that parents could withdraw from the study at any time.

## Results

The study included questionnaire returns from 568 parents of whom 312 (54%) were mothers and 256 (45%) fathers (Table 1). One father had to be excluded because he had more than 20% missing. The response rate for the six hospitals participating in the study varied from 33 to 66%, and the mean was 45% (Table 3). The level of missing data is low (mean 1.1%) for the final survey, which suggests that the questionnaire is acceptable to respondents.

The six hospitals registered the total number of children admitted to their respective care units during the study period, both in total and split according to the variables infant “gestation age” (*GA*) and “length of the stay” (*length-of-stay*). This makes it possible to do a simple comparison of the sample and the targeted population, but only on these two specific variables. The two variables are, however, measured by ordinal scales with very few categories (*GA* has four categories and *length-of-stay* has five categories). This makes it difficult to test the significance of the difference between the sample and the population on these two variables. We are therefore only able to give some rough indication of the representativeness of the sample. This is done by calculating the Spearman rank correlation between the sample and the population on the basis of the two variables. Both the age and the length-of-stay distributions seem to be reasonably represented in the sample. The strongest correlations are found in the three sub-samples unit 1, unit 4, and unit 5 (correlations between 0.9 and 1.00). When taking the above descriptive variables into consideration, the two populations do not appear to differ much from one another.

The mean age of the respondents in the sample is 30.09 years (SD 5.50) for the mothers and 33.10 years (SD 6.94) for the fathers. There was a significant difference of education level between mothers and fathers. More mothers (59%) had a higher education (≥4 years) compared to fathers (46%), and more mothers (30%) were in unpaid work/education compared to fathers (9.4%) (Table 1).

Most parents (94%) characterized their child’s health as good, and 4% characterized it as bad. Out of the 352 infants included in the study, 70% were born with GA ≤ 36.9. The length of stay was from 2 days to about 4 weeks [median, 2 weeks (Table 2)].

### Factor analysis

After running exploratory factor analyse several times, as explained in the method section, eight factors comprising 51 variables (questions) were finally extracted. This final questionnaire with 51 items in eighth factors explained 53.27% of the scale’s variance, and the correlations between questions were acceptable (KMO = 0.938, and the Bartlett’s test of Sphericity gave χ2 = 4813.142. With df = 1275, the significant level of the null hypothesis was far below 0.05 (*P* < 0.0001)).

We further confirmed the reliability/convergent validity of the final version of the NSS (see Table 4). In each factor, the Cronbach’s alpha was above 0.7 for all the items, and the inter-subscale correlation was between 0.70 and 0.94. Average variance extracted (AVE) for the eight factors were mainly above the recommended level of 0.50. The only exception was the *Care and treatment* factor showing an AVE of 0.464. Discriminant validity as present for all the eight factors, as the AVE’s are larger than any of the squared correlations between pair of factors (Tables 4 and 5).

**Table 4:** Exploratory factor analysis and reliability testing results of the NSS-8^a^

Factors	Name of each domain	Number of items	Eigenvalues			Internal reliability	Average variance extracted (AVE)
			Total	% of variance	Cumulative %	Cronbach’s α	
F 1	Care and Treatment	22	9.150	17.942	17.942	0.94	0.464
F 2	Doctors	9	5.898	11.565	29.507	0.91	0.697
F 3	Visit	4	3.118	6.113	35.621	0.91	0.796
F 4	Information	4	2.006	3.933	39.554	0.81	0.679
F 5	Facility	4	1.944	3.811	43.364	0.72	0.552
F 6	Parent anxiety	3	1.835	3.597	46.962	0.74	0.665
F 7	Discharge	3	1.816	3.560	50.522	0.70	0.639
F 8	Siblings	2	1.402	2.749	53.271	0.72	0.736

^a^Varimax rotation

**Table 5:** Squared correlations between the constructs and average variance extracted (on the diagonal)

	F1	F2	F3	F4	F5	F6	F7	F8
F1 Care and treatment	**.464**							
F2 Doctors	.456**	**.697**						
F3 Visit	.286**	.194**	**.796**					
F4 Information	.438**	.370**	.178**	**.679**				
F5 Facility	.267**	.183**	.227**	.145**	**.552**			
F6 Parent anxiety	.040**	.020**	.025**	.027**	.044**	**.665**		
F7 Discharge	.219**	.158**	.085**	.176**	.051**	.012*	**.639**	
F8 Siblings	.254**	.124**	.070**	.115**	.085**	.077**	.083**	**.736**

**Correlation is significant at the 0.01 level (2-tailed). *Correlation is significant at the 0.05 level (2-tailed)

In the table above, we see that all the average variance-extracted values (bold) are larger than any of the squared correlations between pairs of contructs, that is, discriminant validity is established for all constructs

The total alpha of all 51 questions was 0.949. In the corrected item total correlation, the items correlated between 0.362 and 0.718, except for three items concerning questions about the parents’ worries about their child not surviving and after-effects and one item regarding facilities (0.115, 0.136, 0.284). We did not omit these questions because we consider these items to be of clinical importance when measuring the quality of health in NICUs. Pallant (2010) recommends looking at alpha if an item is deleted, and if any of the values in this column are higher than the final alpha value, removing the item [26]. This was not necessary in our analysis. The structure of components, their loadings, the percentages of variance explained by each factor, and the number of items are described in Table 4.

The standardized factor loadings of the items within the factors, from the rotated factor matrix, were as follows: Care and Treatment, 0.709–0.325; Doctors, 0.800–0.325; Visit, 0.806–0.679; Information, 0.713–0.387; Facility, 0.646–0.470; Parent Anxiety, 0.849–0.510; Siblings, 0.818–0.588; and Discharge, 0.635–0.502.

Convergent validity was explored by examining the relationship between the NSS scale and the questions measuring overall satisfaction with care by using the Spearman’s rank correlations (Table 6). Total scores on the NSS were (1) “All in all, how satisfied or dissatisfied are you with the treatment the child/children received at the hospital?” and (2) “All in all, how satisfied or dissatisfied are you with how you as a next of kin were treated?” On overall question 1, 46 of 51 items correlated at the 0.01 significance level. Three items were correlated at the 0.05 significance level, and two questions did not correlate. On overall question 2, 47 items were correlated at the 0.01 significance level, two items were correlated at the 0.05 significance level, and one item did not correlate.

**Table 6:** Convergent validity. Correlation between overall patient satisfaction and components

Factors	Overall item 1	Overall item 2
	Treatment of the infant	Treatment of parents
F1 Care and Treatment	.394**	.514**
F2 Doctors	.323**	.385**
F3 Visit	.229**	.286**
F4 Information	.352**	.393**
F5 Facility	.232**	.263**
F6 Parent anxiety	.135**	.155**
F7 Discharge	.232**	.249**
F8 Siblings	.179*	.162*

Spearman’s rank correlation

** = correlation is significant at the 0.01 level (2-tailed)

* = correlation is significant at the 0.05 level (2-tailed)

The strongest correlation between all-in-all question 1(infant satisfaction) was found in the item “To what extent did you experience that the child/children were taken care of later in the process?” (0.353). The weakest correlation was between overall question 1 and the item “While the child/children were admitted, were you at any time afraid that the child/children would have delayed injury/after-effects?” (0.033).

The strongest correlation found between all-in-all question 2 (parents satisfaction) and the related items was “To what extent did you experience that you were taken care of later in the process?” (0.488). The weakest correlation was between question 2 and the item “During the period of the child’s/children’s admission, were you at any time afraid that the child/children would not survive?” (− 0.028) (table not shown).

Correlations between the 8 factors in the questionnaire and the two global questions of satisfaction provided support for the convergent validity of the questionnaire (Table 6). Factor 1 “Care and Treatment” correlated most strongly with both overall questions (0.394 and 0.514), and factor 6 “Parent Anxiety” had the weakest correlation (0.135 and 0.155) at the 0.01 level. The correlation was significant at the 0.01 level for all components except from component “Siblings” who was significant at the *p* < 0.05 level.

Parents’ satisfaction rates measured in percentage rates for all the eight factors and the two overall quality measures are shown in Table 7. We can see that overall the satisfaction rates are high and SDs are low, suggesting little disagreement in the evaluation of services. The highest satisfaction rate was on factor 1 “Care and Treatment” with a score of 86%, including 22 items. The question “To what extent did you experience that the child/children were taken care of upon arrival in the component at the unit?” scored 95%. Factor 3 “Visit” and factor 5 “Facility” have a shared 2nd place at 85%. The question on factor 5, offering of food/rest, loading, pump room, etc., was the highest item with a score of 89%. The lowest rate was on factor 8 “Siblings”, with a score of 64%. Factor 2 “Doctors” was placed on 4th place, with a score of 81%. The lowest satisfaction rate of all 51 items was in factor 2 on the item “To what extent did you experience that one doctor had the principal responsibility for the child?” with a score of 52%.

**Table 7:** Satisfaction rate with the different areas in NSS-8 (percentage rate)

Factors and global items	Satisfaction rate (%)	St.dev.
F1 Care and treatment	86.16	12.21
F2 Doctors	80.66	16.83
F3 Visit	84.76	16.62
F4 Information	79.92	16.83
F5 Facility	84.89	16.53
F6 Parent anxiety	70.20	22.82
F7 Discharge	75.29	18.67
F8 Siblings	64.43	18.89
Overall quality 1	90.51	23.77
Overall quality 2	86.57	22.94

Satisfaction scores and satisfaction rates for individual items and eight factors were reported. The satisfaction rate was calculated in accordance with the following formula: $\mathrm{Px}=\frac{\left(\mathrm{x}\hbox{--} \kern0.5em -1\right)\times 100}{5-1}=\%\mathrm{satisfaction}\ \mathrm{rate}/\mathrm{item}$

## Discussion

The aim of this study was to validate the NSS-13 and develop a questionnaire that can be used to assess parent satisfaction and experiences with care during the birth of a preterm baby or sick new born hospitalized in a NICU. For full details and comparison of NSS-13 and NSS-8 we refer to Hagen et al. (2015) [18].

After the validating process of NSS-13 the questionnaire was reduced to eight factors (NSS-8). The main findings were that NSS-8 is a suitable instrument to measure parents’ satisfaction in NICUs. Statistical analysis showed that the NSS-8 is valid for its purpose, and the results indicate that the NSS-8 has optimal quality. To prevent form acquiescence bias, we have balanced the items in positively and negatively worded questions. However, early-assessed satisfaction may be influenced by expectations, many mothers gave birth unexpectedly to a premature infant, and this could have a negative impact on satisfaction ratings. It may also be difficult to distinguish between satisfaction with the childbirth experience and satisfaction with the care received. It is therefore suggested to define dimensions of perinatal care, such as staff attitudes and behaviour, information, and environment and make sure that satisfaction can be measured for each individual dimension. This may help refine satisfaction assessment and more accurately delineate aspects of care [27].

The items in the NSS-13 were developed from a comprehensive review of literature, along with existing tools, and based on expert ratings; the content validity of the questionnaire was acceptable. High Cronbach’s alpha and Spearman rank correlation also confirmed the reliability of the questionnaire. There are many different methods to assess construct validity. In this study, we have measured the construct validity of NSS-13 by using exploratory factor analyses and decided on 8 dimensions (NSS-8). The survey was subject to a series of testing processes to assess its reliability and validity. All eight dimensions were similar in some ways to the tools used in previous studies when measuring patient satisfaction with care in hospitals [5, 13, 14, 28–31].

It is not easy to compare the NSS-8 with other instruments of interest, given the aim of the instruments and the different populations. However, some parallels are found between the NSS-8 and other instruments used in similar populations. Bjertnaes et al. (2012) found that the most important predictor for adult patient satisfaction with hospitals in Norway (N63) was the quality of nursing services [29]. Weiss et al. (2009) showed that giving awareness and informing about care and treatment after discharge and paying attention to the parents’ needs for what they want increase their satisfaction with hospital services [32]. Because nurses are probably the most important care providers in hospitals, there should be emphasis on nursing-service quality as one of the determining factors of parents’ satisfaction.

Other studies found a significant relationship between nursing courtesy, respect, careful listening, easy access of care, work environment, and patient-nurse staffing ratio and satisfaction with hospital stay [9, 33–37].

In the present study, the first factor of the questionnaire “Care and Treatment” encompasses items on emotional support, care, assessment when admitted in the unit, and many questions about the nurses. Support from staff is widely recognized as an important factor in measuring satisfaction with health care. The same theme was found by Rafiey et al. (2016) in their survey [38]. It is important for parents to experience a high level of support when admitted to the NICU, and we assume that it will also have a positive influence on their satisfaction with their stay in NICU. Hagen et al. (2015) found that one of the most important factors for the parents’ coping experiences in the NICU was positively influenced when health personnel listened to the parents’ needs and opinions regarding their infant [39]. In our survey, we have items covering this part such as “To what extent did you experience that the care personnel signalled that they had time for you” and “were interested in hearing your opinions as a next of kin”. Similar questions were also found in a Canadian survey [30].

The second factor in our survey is “Doctors”. We can see that some studies also separate doctor’s care from nurse’s care [29, 38], and other studies measure them in the same factor [13, 14, 38]. In Scandinavian hospitals, the nurses are important for the patients and next of kin when measuring satisfaction with care in NICU. Our study showed that satisfaction related to the nurses’ care was the most important, whereas the doctors’ care was ranked on the 4th place (Table 6). These results concurred with those present in other studies in general in Europe [29, 33]. In a Chinese study, doctors ranked first [28].

The third factor was “Visit”. This factor covers items of visit conditions and routines in the unit. In other surveys, we could not find similar items referred to visit. One study from Georgia reported that visit from the infant’s siblings was ranked as one of the least important needs of families of patients in NICUs [40]. In the present survey based on Scandinavian culture, visiting from the infants’ siblings and next of kin is important and was ranked as number three in our satisfaction rate (Table 6).

The fourth factor was “Information”. Receiving good, understandable, and sufficient information is ranked high when measuring quality of health care in hospitals [41, 42]. Schoenfelder et al. found that information did not have a major influence on patient satisfaction [33], but he was exploring patients in hospital, not parents to new-born infant in NICU. The information needs can be different in different settings. Sawyer et al. (2013) identified four key dimensions important when measuring quality in NICU when a premature infant is born: information, explanation, encouragement, and listening to parents with empathy. They included a dimension of information and explanation in their survey covering seven items [14]. We have also covered similar questions in our survey, both in this dimension and spread in some of the others.

The fifth factor is the “Unit Facilities” including both facilities for parents and privacy (patient and next of kin’s physical space in order to avoid intrusive atmosphere and to ensure confidentiality for parents and infants). Questions on facilities were also found in one instrument [28] but were among the least important needs of families of patients in NICUs from both USA and China [28, 40].

The sixth factor is about parent anxiety and two of the four items are also taken from the Groven questionnaire [43]. The remaining two items came from the development of NSS-13 [18]. These questions were important for measuring parent satisfaction when admitted to the NICU; but in Mundy’s study, these items were not regarded as important [40].

Questions about discharge were the seventh factor in our survey, covering three items of receiving necessary information and being prepared to manage the necessary follow-up care of the child after discharge from the NICU. Preparing for discharge was just highlighted in one other study when measuring quality in health care [5].

Questions about siblings were the final factor covering health personnel offering attention to the siblings’ needs and their reaction to parents living in the NICU. This dimension was not found in other studies. Because the hospitalization of a child in NICU will influence the whole family, we consider siblings to be an important aspect to take into account in the questionnaire. The Norwegian government recommends that, if the admitted child has siblings, the unit should provide suitable facilities for them to visit [44]. The clinical implications of this study could help NICUs to monitor parents’ satisfaction and dissatisfaction. NICUs in the western word have much in common. The NSS-8 is designed to measure eight areas associated with satisfaction in NICUs. The different NICUs should compare their structure in order to see if the NSS-8 is suitable in their units. Due to the similarities of NICUs in Scandinavia the generalizability is probable.

## Limitations and strengths

Parents in our study answered the questionnaire while still in the NICU, and they reported overall high satisfaction with their stay. Parents may have felt reluctant to criticize the professionals who had taken care of them and their infant which might have contributed to a bias in questionnaire returns. However, there are reported factors such as long and short perinatal stay, instrumental operative delivery, unexpected medical problems, multiple physical symptoms, and complicated perinatal course that need to be related to dissatisfaction [45]. A longitudinal approach could have been better. Measuring expectations and satisfaction also some time after discharge could have provided a better insight into this phenomenon.

Secondly, the criterion validity using a “gold standard” was not tested. Few validated parent-satisfaction instruments are developed after family-centred care was implemented in NICUs, and we therefore consider “gold standards” not important in our study.

The third limitation is the response rate in this study. The respondents represent 45% of the population but responserate is considrebale higher due to the following explanations, but is higher than average for Norwegian national patient-experience surveys [29]. The lack of data from refugees not speaking any of the Scandinavian languages or conversant with English, was a feature of the survey period, during which parents from Syria formed a notable group. A number of responses were not collected due to administrative errors. The absence of men/fathers during the period of their child’s admission to the NICU is also a consideration. Another reason could be that we had no follow-up or reminder to answer the survey. In our study, 568 parents answered the survey (45%), and this is a large number of respondents from a wide geographical area in Norway. This will also give statistical power and protect from bias. To protect from selection bias, we tested possible differences between the responding and non-responding groups and found no differences in GA and length of stay. However, we cannot predict how the non-responding group would have answered the survey.

Test-retest reliability can assess the stability of a measure over time and is recommended in the process of any questionnaire development. This is of particular importance if the intended use of the measure is to assess change over time or when current mood states are not likely to remain stable over a period of a few weeks.

Finally, we have not considered the possibility of biased sample when both parents have responded to the questionnaire.

The level of missing data is low (mean 1.1%) for the final survey, which suggests that it is acceptable to respondents.

To our knowledge, this survey seems to be the only tool designed for measuring parent satisfaction in NICUs in Scandinavian countries based both on Norwegian/Scandinavian recommendations for measuring quality in health care in hospitals and on experts on health personnel and parents of patients admitted to a NICU.

The NSS-8 is translated to English and is easy to score. The sample size was relatively high for factor analysis, which strengthens the validation process of the questionnaire.

This survey is based on both mother and father, and many fathers participated in the study (45%). This is a strength because studies suggest that the fathers’ experiences with care in NICU differ from those of the mothers [46]. This could therefore influence the fathers’ evaluation of care. Fathers of sick, preterm babies are recognized as a difficult group to recruit for research [47].

## Conclusion

The NSS-8 is a parent-completed survey questionnaire which explores several key aspects of their experiences with NICU. The final 51 questions give good evidence for face and content validity and include important aspects of care in NICU.

The NSS-8 questionnaire is a valid and reliable scale for measuring parent satisfaction with developmental care in NICU. The parent-satisfaction outcomes might contribute to identify interventions to improve the quality of care in NICU and can be used fully or only through a few of the factors. We are confident that NSS-8 will generate insights into different aspects of quality of care, especially in areas where there is a need for improvement, but also to provide an understanding of what is perceived as being done well. We recommend researchers in different countries to further validate the NSS-8.

## Abbreviations

GA: Gestation age
NICU: Neonatal Intensive Care Unit
NSS-13: Neoanatal Satisfaction Survey 13
NSS-8: Neonatal Satifaction Survey 8
